# Short and Long Term Mortality after Coronary Artery Bypass Grafting (CABG) Is Influenced by Socioeconomic Position but Not by Migration Status in Sweden, 1995–2007

**DOI:** 10.1371/journal.pone.0063877

**Published:** 2013-05-22

**Authors:** Dashti Ali M. Dzayee, Torbjörn Ivert, Omid Beiki, Lars Alfredsson, Rickard Ljung, Tahereh Moradi

**Affiliations:** 1 Institute of Environmental Medicine, Division of Epidemiology, Karolinska Institutet, Stockholm, Sweden; 2 Departments of Cardiothoracic Surgery and Anesthesiology, Karolinska University Hospital and Molecular Medicine and Surgery, Karolinska Institutet, Stockholm, Sweden; 3 Kermanshah University of Medical Sciences, Kermanshah, Iran; 4 Unit of Clinical Epidemiology, Department of Medicine, Karolinska Institutet, Stockholm, Sweden; 5 Centre for Occupational and Environmental Medicine, Stockholm County Council, Stockholm, Sweden; 6 Unit of Upper Gastrointestinal Research, Department of Molecular Medicine and Surgery, Karolinska Institutet, Stockholm, Sweden; 7 Equity and Health Policy, Department of Public Health Sciences, Karolinska Institutet, Stockholm, Sweden; 8 Centre for Epidemiology and Social Medicine, Healthcare Provision, Stockholm County, Stockholm, Sweden; University of Adelaide, Australia

## Abstract

**Background:**

There are no nationwide studies on mortality after coronary artery bypass grafting (CABG) among foreign-born populations that include detailed information about country of birth and information about socioeconomic position. The objective was to investigate the risk of mortality after CABG considering socioeconomic position, sex and country of birth.

**Material and Methods:**

We included all 72 333 patients undergoing a first isolated CABG in Sweden, during 1995 - 2007 of whom 12.7% were foreign-born. The patients were classified according to educational level, sex, and country of birth and were followed up to December 2007. We estimated the risk of short and long term mortality after CABG in a multivariable model adjusted for age, calendar year of surgery, diabetes, educational level, and waiting time for surgery. Hazard ratios (HR) with 95% confidence intervals (CI) were calculated based on the Cox proportional hazard model.

**Findings:**

There were 15,284 deaths during the follow-up, 10.4% of whom were foreign-born. The foreign-born patients were 3 to 4 years younger than Sweden-born patients at the time of CABG surgery. There were no significant differences in overall early or late mortality between foreign-born and Sweden-born men and women after CABG. All-cause mortality differed in between regions and was highest in foreign-born men from Eastern Africa (HR 3.80, 95% CI 1.58–9.17), China (HR 3.61, 95% CI 1.50–8.69), and in Chile (HR 2.12, 95% CI 1.01–4.47). Patients with low level of education had worse survival compared to those with longer than 12 years of education irrespective of sex and country of birth. This difference was more pronounced among foreign-born women (HR 1.50, 95% CI 1.00–2.33).

**Conclusion:**

This national study showed higher CABG mortality in patients from lower socioeconomic position. Early and late mortality did not differ after isolated CABG in foreign-born and Sweden-born patients.

## Introduction

Atherosclerosis is the major underlying cause responsible for adverse cardiovascular outcome including transient ischemic attack, stroke, and coronary arterial disease (CAD) [1]. It is a chronic process and with the configurations of the plaque formation and atherosclerotic stenosis can lead to final plaque rupture [2], [3], [4], [5]. In recent years, a variety of advanced techniques in imaging modalities, including magnetic resonance imaging, trans-esophageal echocardiography, and computed tomographic angiography have been introduced in the assessment of CAD [6], [7]. To treat symptoms of CAD, coronary artery bypass grafting (CABG) was introduced in late 1960s [8]. It is an effective method to relieve symptoms of angina pectoris and to improve function and survival in a relatively high proportion of patients with ischemic heart disease (IHD) [9], [10], [11]. In industrialized countries including Sweden, CABG has been a common procedure. However, due to the advent of percutaneous coronary interventions (PCI) during the last decade, a decline in the number of CABG has been observed [12], [13], [14]. A few reports have explored differences in access to invasive cardiac procedures in relation to ethnic, socioeconomic and sex disparities worldwide [15], [16], [17], [18]. Difference in survival after CABG in men and women have been reported [19], [20], [21], [22], [23], but the influence of country of birth and socioeconomic position (SEP) has not been fully investigated. We evaluated short and long-term mortality after CABG in Sweden in relation to SEP, sex and country of birth using a variety of Swedish national registers.

## Materials and Methods

### Ethics Statement

By Swedish law data recording to national health data registers, like the Patient Register, do not need consent from the patients, nor from the health care providers. It is mandatory to report to these registers, and the patients cannot decline registration. The quality health care registers, like the Heart Surgery Register, are voluntary and the patients need to be informed about the register, but they do not need to give written or oral consent to be recorded. However, patients can at any time decide to not allow registration in these quality health care registers. Ethical vetting is always required when using register data for research in Sweden. The ethical vetting is performed by regional ethical review boards and the risk appraisal associated with the Law on Public Disclosure and Secrecy is done by data holders. The ethical review boards can however waive the requirement to consult the data subjects directly to obtain their informed consent. According to these standards the Regional Ethical Review Board in Stockholm, Sweden, has waived the requirement to consult the data subjects directly to obtain their informed consent for this project and has evaluated and approved the project.

### Study Population and Follow Up

The study cohort consisted of all 72 333 individuals who underwent a first isolated CABG from January 1995 to December 2007 in Sweden of whom 12.7% were foreign-born. A vast majority of the operations were performed with the aid of cardiopulmonary bypass but during 1998 up to 2002 about 10% of the patients were operated without extracorporeal circulation. The subjects were followed from the date of CABG until the date of death from any cause, date of emigration or end of follow up at December 31, 2007, whichever came first.

### Linkage to Swedish National Registers

The data used in our study were collected by linkage of several public national registers through Swedish unique individual 10-digit personal identity number; are based on the Migration and Health Cohort [24] specifically designed to address health status among immigrants in Sweden. We used part of Migration and Health Cohort as follows:

1. The Swedish Heart Surgery Registry, Swedish Coronary Angiography and Angioplasty Registry (SCAAR) and the Register of Information and Knowledge about Swedish Heart Intensive Care Admissions (RIKS-HIA) [25]. More than 7000 heart surgery procedures including CABG were included yearly with demographic data, information on type of operation, certain postoperative complications and risk variables according to Euro SCORE (European System for Cardiac Operative Risk Evaluation). SCAAR and RIKS-HIA provided information of PCI and admittance to coronary care units, respectively.
2. The Register of the Total Population at Statistic Sweden containing demographic information, country of birth and data on emigration and immigration [26].
3. The Cause of Death Register with information on date of death and underlying and contributing cause of death since 1952 [27].
4. The Swedish Population and Housing Census and the longitudinal integration database for health insurance and labor market studies (LISA) where information on highest acquired level of education was retrieved [28].

### Patients Characteristics and Covariates

Information on previous medical history including diabetes mellitus was classified according to the International Classification of diseases, ICD 9 and ICD 10. Waiting time for surgery defined as the time since indication to operation was categorized into five categories (emergently within 24 hrs., 1–6 days, 7–30 days, 31–90 days, and longer than 90 days). Many variables such as smoking and body mass index, as well as left ventricular function, number of vessels diseased, severity and indication of the surgery were not possible to include because of large number of missing information.

#### Socioeconomic position

We used highest attained level of education as an indicator for SEP. Level of education was divided into four categories: 0–9 years (compulsory school education), 10–12 years (high school education), more than 12 years (university and highest level of education), and unknown.

#### Age and calendar years

Age at surgery was divided into five groups (younger than 50, 50–59, 60–69, 70–79, and 80 years and older). The year of surgery was divided into three intervals through the total study period: 1995–1998, 1999–2002, and 2003–2007.

#### Country of birth

We classified foreign-born individuals according to six continents, which were subdivided into 19 world regions, as defined by the United Nations Population Division: Africa (Eastern, Central, Northern, Southern and Western Africa), Asia (Eastern, South-Central, South-Eastern and Western Asia), Europe (Eastern, Northern, Southern and Western Europe), Latin America (Caribbean, Central America and South America), North America and Oceania (Australia/New Zealand, Melanesia, Micronesia/Polynesia). We reported results regarding birth country for all continents and regions. For individual countries results were reported if the number of fatal cases was five or more.

### Statistical Methods

We used Cox Proportional Hazards model to estimate the relative rate of death after CABG. Hazard ratios (HR) and 95% confidence intervals (CI) were calculated using the maximum partial likelihood for the effect estimates. In the analysis of mortality according to country of birth, Swedish born were used as reference category. When analyzing education, highest attained level of education was reference. All analyses were performed separately for men and women, adjusted for age at surgery, calendar year of surgery, waiting time for surgery, diabetes mellitus, and educational level. The assumptions of Cox regression were met. We used SAS version 9.2 for all analyses. We considered s *P*-value of <0.05 as statistically significant and all tests were two-sided.

## Results

### Study Population

During the 13-year observation period (median 5.3, range 0–12.9 years), we recorded 15, 284 deaths among 72, 333 patients who underwent a first isolated CABG (Table 1). A total of 1, 588 of the deaths (10.4%) occurred among the foreign-born patients. Although average age in both groups increased during three time intervals, foreign-born patients were 3 to 4 years younger than Sweden-born patients during the entire study period. Age more than 70 years at the time of surgery was less common among foreign-born but increased in more recent years. Female sex was more common among foreign-born patients. Diabetes mellitus was more prevalent in more recent years in both groups. Irrespective of country of birth, few patients were operated on emergently within 24 hrs. Waiting time for surgery longer than three months decreased in both groups in recent years. A somewhat higher proportion of patients with higher level of education were observed among foreign-born individuals.

**Table 1:** Characteristics of patient undergoing coronary artery bypass grafting in relation to time period of the operation and migration status, Sweden 1995–2007.

	1995–1998†N (%)		1999–2002†N (%)		2003–2007†N (%)			
	Foreign-born2649 (11)	Sweden-born20601 (89)	Foreign-born3005 (12)	Sweden-born21092 (88)		Foreign-born3523 (14)	Sweden -born21463 (86)	
**Age**	62±9	66±9	63±9	67±9	64±9		67±9	
**Sex**								
**Male**	1920 (72)	15568 (76)	2188 (73)	16169 (77)	2577 (73)		16564 (77)	
**Female**	729 (28)	5033 (24)	817 (27)	4923 (23)	946 (27)		4899 (23)	
**Diabetes**								
**Yes**	456 (17)	2941 (14)	708 (23)	3888 (18)	936 (27)		4457 (21)	
**No**	1897(72)	14687 (71)	2245 (75)	16761 (80)	2582 (73)		16922 (79)	
**No information**	296(11)	2973 (15)	52 (2)	443 (2)	5 (0)		84 (0)	
**Waiting time**								
**<24 hrs**	88 (3)	497 (2)	56 (2)	485 (2)	122 (3)		736 (3)	
**1–6 days**	397 (15)	3408 (17)	588 (19)	4437 (21)	877 (25)		5298 (25)	
**7–30 days**	510 (19)	3740 (18)	744 (25)	4984 (24)	1183 (34)		6798 (32)	
**31–90 days**	792 (30)	6123 (30)	814 (27)	5445 (26)	798 (22)		5178 (24)	
**≥90 days**	575 (22)	4535 (22)	531 (18)	3403 (16)	418 (12)		2470 (11)	
**No information**	287 (11)	2298 (11)	272 (9)	2338 (11)	125 (4)		985 (5)	
**Education**								
**Unknown**	184 (6)	111 (1)	287 (7)	91 (0)	287 (8)		58 (0)	
**0–9**	1227 (46)	11434 (55)	1249 (42)	10796 (52)	1377 (39)		10106 (48)	
**10–12**	891 (34)	6586 (32)	1103 (37)	7377 (35)	1281 (36)		894 (37)	
**>12**	347 (14)	2470 (12)	479 (14)	2828 (13)	578 (17)		3285 (15)	

† Number patients.

### Main Causes of Death after CABG by Country of Birth

The main causes of death were IHD followed by malignancy and cerebrovascular accident (CVA) during the study period (Table 2). The percentage of early and late deaths from IHD was slightly higher in foreign-born than in Sweden-born patients.

**Table 2:** Characteristics of patient undergoing coronary artery bypass grafting in relation to causes of death since operation and migration status, Sweden 1995–2007.

			Main cause of death				
		IHD†No. (%)	Cancer†No. (%)	CVA†No. (%)	*Other causes†No. (%)	Total†No. (%)	γP value
**30 Days**	**Foreign-born**	118 (65.19)	0	3 (1.66)	60 (33.15)	181(%100)	0.73
	**Sweden-born**	841 (62.95)	4 (0.30)	43 (3.22)	448 (33.53)	1336(%100)	
**1 Year**	**Foreign-born**	198 (54.25)	21 (5.75)	21 (5.75)	125 (34.25)	365(%100)	0.85
	**Sweden-born**	1446 (52.56)	171 (6.22)	142 (5.16)	992 (36.06)	2751(%100)	
**5 years**	**Foreign-born**	393 (42.86)	183 (19.96)	58 (6.32)	283 (30.86)	916(%100)	0.01
	**Sweden-born**	3021 (40.30)	1492(19.90)	584 (7.79)	2400 (32.01)	7497(100%)	
**All period**	**Foreign-born**	631 (39.74)	323 (20.34)	117 (7.37)	517 (32.56)	1588(%100)	<0 001
	**Sweden-born**	5156 (37.65)	2806 (20.49)	1136 (8.29)	4598 (33.56)	13696(%100)	

† Number of event.

γ P value refers to distribution of main cause of death between Sweden-born and Foreign-born.

* Other causes of death included infectious diseases and trauma.

### All-cause Mortality after CABG by Socioeconomic Position

Patients with low level of education had statistically significantly worse survival compared with those with longer than 12 years of education irrespective of sex and country of birth. This difference was more pronounced among foreign-born women (HR 1.53, 95% CI 1.00–2.33) (Tables 3).

**Table 3:** Hazard ratio (HR) and 95% confidence interval (CI) of mortality after CABG among men in Sweden, 1995–2007.

	Foreign-bornmen			Sweden-bornmen	
	Event		HR* (95% CI)	Event	HR* (95% CI)
Years ofeducation					
**Unknown**	121		**1.77 (1.37–2.28)**	72	**1.70 (1.34–2.16)**
**0–9**	489		**1.39 (1.14–1.70)**	5839	**1.29 (1.21–1.38)**
**10–12**	384		**1.26 (1.03–1.54)**	3195	**1.16 (1.08–1.24)**
**>12**	126		1	1114	1
	**Foreign-born women**			**Sweden-born women**	
**Unknown**	44	**1.20 (0.73–1.99)**		46	**1.65 (1.19–2.27)**
**0–9**	301	**1.53 (1.00–2.33)**		2326	**1.28 (1.11–1.47)**
**10–12**	99	1.09 (0.69–1.71)		884	1.13 (0.97–1.31)
**>12**	24	1		220	1

* Adjusted for age, calendar year of surgery, waiting time for operation, and diabetes mellitus.

### All-cause Mortality after CABG by Sex

Overall, men had a worse survival than women after multivariable adjustment (HR 1.14, 95% CI 1.10–1.18) (results not shown in the table).

### All-cause Mortality after CABG by Country of Birth

**Figure 1:**
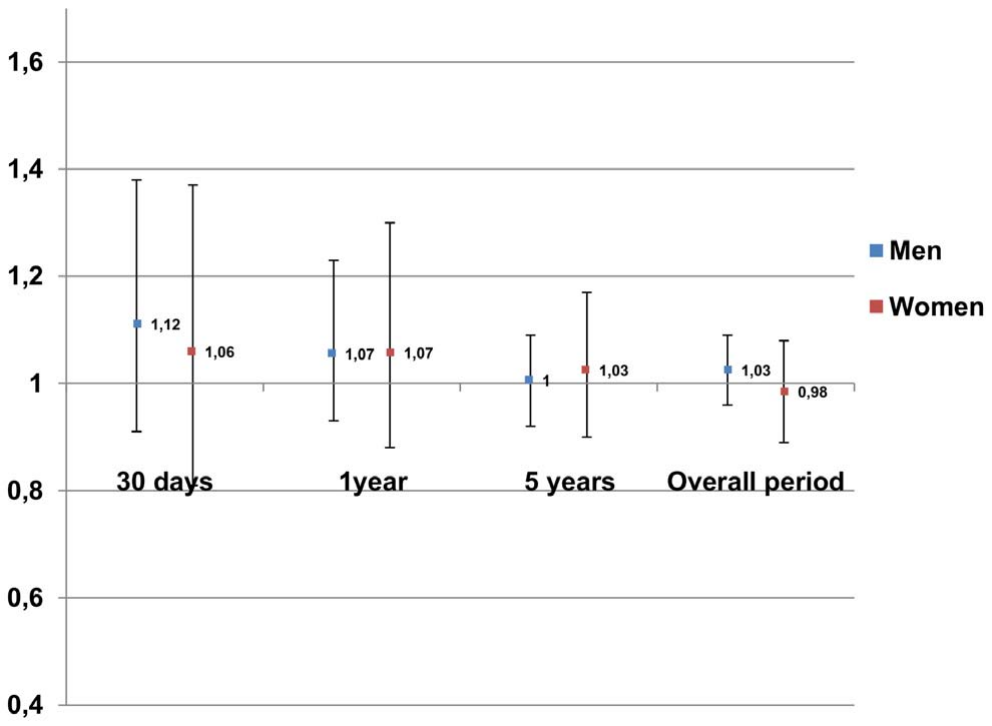
Hazard ratio (HR) and 95% confidence interval (CI) of mortality after CABG among Foreign-born compared to Sweden-born in Sweden, 1995–2007. Hazard ratio is adjusted for age, calendar year, waiting time for operation, diabetes mellitus, and education.

There were no significant differences in the risk of early or late death after the operation between foreign-born and Sweden-born men and women throughout the study period (Table 4). In foreign-born men the overall risk was 7% higher compared to Sweden-born men after adjustment for age at operation and year of surgery but no significant difference was found after multivariable adjustment. There was a slight improvement in overall survival among foreign-born compared to Sweden-born (Figure 1).

**Table 4:** Hazard ratio (HR) and 95% confidence interval (CI) of mortality after CABG among Foreign-born compared to Sweden-born in Sweden, 1995–2007.

	Men		Women	
Period sinceoperation	HR†(95% CI)	HR* (95% CI)	HR†(95% CI)	HR* (95% CI)
**30 days**	1.14 (0.94–1.40)	1.12 (0.91–1.38)	1.07 (0.83–1.38)	1.06 (0.81–1.37)
** 1year**	1.11 (0.97–1.27)	1.07 (0.93–1.23)	1.09 (0.90–1.31)	1.07 (0.88–1.30)
** 5 years**	1.01 (0.93–1.10)	1.00 (0.92–1.09)	1.03 (0.91–1.17)	1.03 (0.90–1.17)
**Overall** **Period**	1.07 (1.00–1.14)	1.03 (0.96–1.09)	1.02 (0.92–1.12)	0.98 (0.89–1.08)

† Adjusted for age and calendar year of surgery.

* Adjusted for age, calendar year of surgery, waiting time for operation, diabetes mellitus, and education.

Older age at operation was associated with worse survival regardless of sex. Patient with diabetes mellitus or missing information of diabetes had worse survival and had higher risk of death than patients without diabetes mellitus with hazard ratios (men: foreign-born: HR 1.60, 95% CI 1.40–1.84; Sweden-born: HR 1.64, 95% CI 1.56–1.72) and (women: foreign-born: HR 1.53, 95% CI 1.24–1.88; Sweden-born: HR 1.84, 95% CI 1.70–1.99). Apart from those who underwent acute operation, waiting time for surgery did not correlate to mortality after CABG in our study (results not shown).

### All-cause Mortality after CABG by Continent and Specific Country of Birth

Considering continent and region of birth, men born in Europe, Southern Europe, Eastern Asia and men and women born in Eastern Africa had higher risk of death after CABG compared with Sweden-born men and women (Tables 5 and 6). In contrast, men born in Asia and South-Central Asia, showed lower risk compared with Sweden-born individuals. At the country level, higher relative risks were found among men born in Denmark, Bosnia, Chile, and men and women born in China. Men born in Iran showed lower risk compared with men born in Sweden.

**Table 5:** Hazard ratio (HR) and 95% confidence interval (CI) of mortality after CABG among men by country of birth in Sweden, 1995–2007.

Birth Region/Country	Event†	HR* (95% CI)	Birth Region/Country	Event†	HR* (95% CI)
Sweden-born	10 220	**1(ref)**			
Africa	15	1.47 (0.88–2.44)	**Europe**	976	**1.07 (1.00–1.15)**
*Eastern Africa*	5	**3.80 (1.58–9.17)**	***Eastern Europe***	135	1.07 (0.90–1.27)
*Middle Africa*	1	N/A	^∧^Other	3	N/A
			Ex- Czechoslovakiaγ	21	1.11 (0.72–1.71)
*Northern Africa*	7	1.05 (0.50–2.20)	Hungary	34	1.09 (0.77–1.52)
*Southern Africa*	2	N/A	Poland	49	1.17 (0.88–1.55)
*Western Africa*	-	N/A	Romania	7	0.74 (0.35–1.57)
Asia	100	0.79 (0.64–0.97)	Ex-Soviet Unionγ	21	0.92 (0.60–1.42)
*Eastern Asia*	5	**3.26 (1.35–7.85)**	***Northern Europe***	588	1.06(0.97–1.15)
**China**	5	**3.61 (1.50–8.69)**	Denmark	116	**1.39 (1.16–1.68)**
*South-Central Asia*	24	0.50 (0.34–0.76)	Estonia	34	0.93 (0.66–1.30)
**Iran**	20	0.58 (0.37–0.91)	Finland	333	1.00 (0.89–1.11)
**Other**	4	N/A	Latvia	6	1.55 (0.69–3.45)
*South-Eastern Asia*	4	N/A	Norway	94	1.07 (0.87–1.31)
*Western Asia*	67	0.94 (0.73–1.21)	UK	5	0.80 (0.33–1.94)
**Iraq**	20	0.89 (0.57–1.40)			
**Lebanon**	8	0.78 (0.38–1.56)	***Southern Europe***	151	**1.26 (1.07–1.49)**
**Syria**	7	0.65 (0.31–1.37)	Bosnia	61	**1.71 (1.31–2.23)**
**Turkey**	24	1.09 (0.73–1.64)	Italy	14	1.10 (0.65–1.87)
**^∧^Other**	8	1.79 (0.89–3.60)	Ex-Yugoslaviaγ	63	1.22 (0.95–1.57)
			Greece	10	0.73(0.39–1.37)
			^∧^Other	3	N/A
			***Western Europe***	102	0.98(0.81–1.19)
			Austria	11	0.59 (0.33–1.08)
			Germany	79	1.13 (0.91–1.42)
			Netherlands	8	0.92 (0.46–1.85)
			^∧^Other	4	N/A
			**Latin America**	12	0.89 (0.51–1.58)
			***Caribbean***	1	N/A
			***South America***	11	0.95 (0.53–1.73)
			Chile	7	**2.12 (1.01–4.47)**
			Other	4	N/A
			**Northern America**	16	0.85 (0.52–1.39)
			USA	16	0.97 (0.59–1.58)
			**Oceania**	1	N/A

* Adjusted for age, calendar year of surgery, waiting time for operation, diabetes mellitus, and education.

γ Ex-Czechoslovakia includes Czech Republic and Slovakia. Ex-Soviet Union includes Belarus, Moldova, Russian Federation and Ukraine. Ex-Yugoslavia includes Croatia, Macedonia, Serbia, Slovenia and Montenegro.

† all causes of death. ^∧^countries with fewer than five event N/A not applicable with less with fewer than 5 events.

**Table 6:** Hazard ratio (HR) and 95% confidence interval (CI) of survival after CABG among women by country of birth in Sweden, 1995–2007.

Birth Region/Country	Event†	HR* (95% CI)	Birth Region/Country	Event†	HR* (95% CI)
Sweden-born	3 476	**1(ref)**			
Africa	3	N/A	**Europe**	415	1.02 (0.92–1.13)
			***Eastern Europe***	64	1.14 (0.89–1.47)
			Ex- Czechoslovakiaγ	11	**1.80 (0.99–3.26)**
			Hungary	11	1.20 (0.66–2.16)
			Poland	27	1.03 (0.70–1.51)
			Romania	5	1.25 (0.52–3.01)
Asia	39	0.78 (0.56–1.10)	Ex-Soviet Unionγ	10	0.96 (0.51–1.81)
*Eastern Asia*	3	N/A	***Northern Europe***	271	0.98 (0.87–1.11)
			Denmark	27	1.00 (0.68–1.47)
*South-Central Asia*	14	0.84 (0.49–1.43)	Estonia	11	1.39 (0.77–2.52)
**Iran**	12	0.97 (0.55–1.74)	Finland	177	0.93 (0.80–1.09)
**^∧^Other**	2	N/A			
*South-Eastern Asia*	2	N/A	Norway	53	1.12 (0.85–1.47)
*Western Asia*	20	0.69 (0.44–1.10)			
			^∧^Other	3	N/A
			***Southern Europe***	35	1.07 (0.75–1.51)
**Syria**	5	0.95 (0.39–2.31)	Bosnia	16	1.33 (0.79–2.24)
**Turkey**	8	0.76 (0.38–1.54)			
**^∧^Other**	7	0.54(0.25–1.16)	Ex-Yugoslaviaγ	17	1.01 (0.62–1.64)
			^∧^Other	2	N/A
			***Western Europe***	45	1.06(0.79–1.42)
			Austria	6	1.73 (0.78–3.87)
			Germany	35	0.96 (0.69–1.34)
			^∧^Other	4	N/A
			**Latin America**	3	N/A
			**Northern America**	7	1.17 (0.55–2.46)
			^∧^Other	2	N/A
			USA	5	1.11 (0.46–2.69)
			**Oceania**	1	N/A

* Adjusted for age, calendar year of surgery, waiting time for operation, diabetes mellitus, and education.

γ Ex-Czechoslovakia includes Czech Republic and Slovakia. Ex-Soviet Union includes Belarus, Moldova, Russian Federation and Ukraine. Ex-Yugoslavia includes Croatia, Macedonia, Serbia, Slovenia and Montenegro.

† all causes of death. ^∧^countries with fewer than five event N/A not applicable with less with fewer than 5 events.

The highest risks were found in foreign-born men born in Eastern Africa (HR 3.80, 95% CI 1.58–9.17), China (HR 3.61, 95% CI 1.50–8.69), and in Chile (HR 2.12, 95% CI 1.01–4.47). (Tables 5 and 6).

## Discussion

In this nation-wide cohort study, low socioeconomic position was independently of sex and country of birth associated with higher mortality after CABG during our entire observation period. However, neither early nor late mortality was influenced by migration status.

The large study cohort including all patients who had undergone CABG in Sweden with long and complete follow-up, the complete nationwide coverage of exposure (country of birth) and outcome (death) were unique characteristics of our study.

Limitations of the study were having insufficient number of observations for valid analyses regarding important life style factors such as smoking and body mass index, condition such as left ventricular function, number of diseased vessels, completeness of revascularization, indication for surgery and repeat revascularization. During 1995 up to 1998 information of diabetes was missing in more than one of ten patients. Furthermore medical management after hospital discharge was not known.

If we could adjust for unmeasured life style factors the increased relative risk for foreign-born would probably be lower, as we anticipate, among others, smoking to be more common among foreign-born men than among Swedish men [29], [30]. For this study we did not classify the foreign-born as refugees, or labor immigrants. However, it has been shown that there are differences in mortality from cardiovascular disease between refugees and non-refugee immigrants [31], [32].

The association we identified between low SEP and poor survival after CABG is in agreement with findings in previous studies that have found SEP to be an important risk indicator for cardiovascular diseases (CVD) in general and CABG mortality in particular [16], [33], [34], [35], [36], [37], [38], [39]. In Sweden with good economy and well-functioning national health coverage such finding is surprising. In Sweden CABG is centralized and performed at eight University Hospitals without large variation in volume and no differences in quality between these units [40], [41]. Indication for CABG is based on patient symptoms and risk factors regardless of ethnicity, SEP or gender. In the US low socioeconomic status and low CABG volume has been correlated to higher mortality [34].

However, a strong influence of SEP on access to specialized cardiac services in Canada with known universal health coverage has also been observed [16]. Unaccounted confounding by life style factors could be one explanation, however, adjustment for diabetes, which is more common among foreign-born and education, did not change the results in this study. The association of low SEP with poor clinical risk factors and CVD biomarkers [39], [42] and with higher prevalence of CHD risk factors [43], [44], [45] could partially explain the observed increased mortality after CABG surgery among patients with lowest SEP in our study.

Men showed higher risk compared to women. The findings regarding short-term mortality in other settings have been contradictory [46], [47], [48], [49], [50], [51]. However, long term all-cause mortality has been shown to be higher among men compared with women [52].

Our finding of no significant survival differences in foreign-born compared with Sweden-born men and women could partially be explained by the universal Swedish health system and that Sweden is a welfare state with relatively low health inequity [53], [54]. A decision to perform CABG should be based on symptoms and extent of coronary artery pathology. However, a higher rate of adverse outcome after CABG among minorities has been shown in the United States even after adjustment of important predictors of death after CABG [55].

### Conclusions

This large and complete nationwide cohort study indicates that low socioeconomic position was independently of sex and country of birth associated with higher mortality after coronary artery bypass grafting (CABG) during the entire observation period in our study. Furthermore, neither early nor late mortality is influenced by migration status.
